# Impact of 2009 American Recovery and Reinvestment Act (ARRA) health center investments on disadvantaged neighborhoods after recession

**DOI:** 10.1186/s13561-024-00482-x

**Published:** 2024-01-31

**Authors:** Elizabeth L. Tung, Nour Asfour, Joshua D. Bolton, Elbert S. Huang, Calvin Zhang, Luc Anselin

**Affiliations:** 1 Section of General Internal Medicine, Department of Medicine, University of Chicago, Chicago, USA; 2 Center for Health and the Social Sciences, University of Chicago, Chicago, USA; 3 Center for Spatial Data Science, University of Chicago, Chicago, IL USA; 4 Bureau of Primary Health Care at the U.S. Department of Health and Human Services, Health Resources and Services Administration (at the time of authorship), Rockville, MD USA; 5 Center for Chronic Disease Research and Policy, University of Chicago, Chicago, IL USA

**Keywords:** Neighborhood disadvantage, Neighborhood socioeconomic disadvantage, Federally-qualified health centers, Recession, Local economies

## Abstract

**Background:**

Federally qualified health centers (FQHCs) are integral to the U.S. healthcare safety net and uniquely situated in disadvantaged neighborhoods. The 2009 American Recovery and Reinvestment Act (ARRA) invested $2 billion in FQHC stimulus during the Great Recession; but it remains unknown whether this investment was associated with extended benefits for disadvantaged neighborhoods.

**Methods:**

We used a propensity-score matched longitudinal design (2008–2012) to examine whether the 2009 ARRA FQHC investment was associated with local jobs and establishments recovery in FQHC neighborhoods. Job change data were obtained from the Longitudinal Employer-Household Dynamics (LEHD) survey and calculated as an annual rate per 1,000 population. Establishment change data were obtained from the National Neighborhood Data Archive (NaNDA) and calculated as an annual rate per 10,000 population. Establishment data included 4 establishment types: healthcare services, eating/drinking places, retail establishments, and grocery stores. Fixed effects were used to compare annual rates of jobs and establishments recovery between ARRA-funded FQHC census tracts and a matched control group.

**Results:**

Of 50,381 tracts, 2,223 contained ≥ 1 FQHC that received ARRA funding. A higher proportion of FQHC tracts had an extreme poverty designation (11.6% vs. 5.4%), high unemployment rate (45.4% vs. 30.3%), and > 50% minority racial/ethnic composition (48.1% vs. 36.3%). On average, jobs grew at an annual rate of 3.84 jobs per 1,000 population (95% CI: 3.62,4.06). In propensity-score weighted models, jobs in ARRA-funded tracts grew at a higher annual rate of 4.34 per 1,000 (95% CI: 2.56,6.12) relative to those with similar social vulnerability. We observed persistent decline in non-healthcare establishments (-1.35 per 10,000; 95% CI: -1.68,-1.02); but did not observe decline in healthcare establishments.

**Conclusions:**

Direct funding to HCs may be an effective strategy to support healthcare establishments and some jobs recovery in disadvantaged neighborhoods during recession, reinforcing the important multidimensional roles HCs play in these communities. However, HCs may benefit from additional investments that target upstream determinants of health to mitigate uneven recovery and neighborhood decline.

**Supplementary Information:**

The online version contains supplementary material available at 10.1186/s13561-024-00482-x.

## Introduction

The American Recovery and Reinvestment Act (ARRA) of 2009 was an $831 billion stimulus package in response to the Great Recession that included several provisions for healthcare [1, 2]. The goal of ARRA was to save existing jobs, create new jobs, and also prevent further deterioration of critical economic sectors such as healthcare. Provisions included tax relief for families, spending on infrastructure projects, and investments in education, energy, homeland security and healthcare [1, 2]. While some better-known healthcare investments included the Health Information Technology for Economic and Clinical Health (HITECH) Act and state aid for Medicaid, the package also appropriated $2 billion in direct funding to Federally Qualified Health Centers (FQHCs) supported by the Health Resources and Services Administration (HRSA) [1, 3]. Although this was a relatively small proportion of overall healthcare investments, the health center provision was unique, because FQHCs provide community-based healthcare to some of the most medically underserved and disadvantaged communities in the United States.

FQHCs are federally funded health centers that have been a focal point of bipartisan U.S. policy regarding disadvantaged communities since their inception in the 1960s. These FQHCs provide a central place for medically underserved communities to receive primary care regardless of their ability to pay. Through federal grant funding, the HRSA Health Center Program aims to support FQHCs that improve the health of geographically isolated, economically vulnerable, or medically vulnerable populations [4]. Currently, HRSA funds nearly 1,400 FQHCs across all 50 United States [4]. Few other ARRA provisions specifically targeted disadvantaged communities in this way. Previous literature has documented that overall ARRA funding may have even benefited high-income areas more than low-income areas [5, 6]. In one study, Gimpel and colleagues estimated that areas in the 90th percentile for median income received $21 per capita *more* than those in the 10th percentile [5].

Importantly, FQHCs and their surrounding communities often experience severe hardship during and after recession [7–9], a process that has recurred at least 13 times since the 1940s [10]. Specifically, growth in uninsured populations due to job loss is a common challenge that safety net providers must face during economic downturns. Between 2007 and 2009, 8.3 million Americans lost their employer-sponsored insurance, adding to the 50 million uninsured Americans living in the United States [11]. To make matters worse, Medicaid expansion was not widely adopted until 2014 and was initially only implemented in a subset of states, leaving millions of poor and uninsured Americans in the “coverage gap” for many years following recession [12]. FQHCs thus played a unique role during the Great Recession by providing care to the growing number of Americans that became uninsured or underinsured. Felland and colleagues examined the financial health of safety net providers in five urban communities after the recession [13]. The authors noted that while these FQHCs appeared to have survived the economic downturn, numerous financial challenges were ongoing even in 2010, corroborating how severely the economic downturn impacted these health centers [13]. 

Beyond safety net care, U.S. health centers may also play a key supportive role in their local neighborhood economies. In some neighborhoods, FQHCs function to provide consistent employment and economic opportunity to local residents. At Lawndale Christian Health Center in Chicago, for instance, a core tenant of the health center’s mission is to redistribute resources to local neighborhoods, which includes employing community residents [14]. While this may be the mission of many FQHCs, no studies to our knowledge have examined whether this stimulus provided to FQHCs had extended benefits for the local economies of disadvantaged neighborhoods. Even fewer studies have examined healthcare establishments and how their recovery may have differed from other neighborhood establishments, such as grocery and retail establishments.

The purpose of this study was to examine the impact of the 2009 ARRA health center investment on local jobs and establishments recovery in a health center’s immediately surrounding community. Importantly, recent debates [15] about stimulus in response to the COVID-19 pandemic and threats of looming recession have led policy makers to revisit previous ARRA provisions, aiming to identify evidence-based opportunities to advance current funding efforts, inform future funding efforts, and improve the long-term recovery of high-poverty communities. The 2009 ARRA health center provision, in particular, emphasized “shovel-ready” projects, allocating $1.5 billion to physical infrastructure projects, including construction and expansion of health centers, and $500 million to operational/service needs [2]. We re-examined this 2009 funding effort, hypothesizing that direct investment in U.S. FQHCs during recession may have had extended economic benefits to surrounding disadvantaged neighborhoods. Evidence is direly needed to improve our understanding of strategies that may more equitably distribute economic recovery to disadvantaged neighborhoods. Leveraging existing healthcare programs and services embedded in underserved communities may be a missed opportunity for impact.

## Methods

We implemented a propensity-score matched longitudinal panel design (2008–2012) to examine the effects of ARRA health center funding on local jobs and establishments recovery in FQHC neighborhoods. We selected this study period because under the Affordable Care Act, another $11 billion was appropriated to health centers in 2011, with the bulk of funding made available by 2013 [3]. Addresses for each FQHC site that received a capital development grant were geocoded to the census tract level. The U.S. Census Bureau uses census tracts to define small, relatively permanent statistical subdivisions of a county, with the population of each census tract ranging from 1,200 to 8,000 residents [16]. 

Census tracts containing ARRA-funded FQHCs were compared to a propensity score-weighted control group of remaining census tracts in the same county. A propensity score match was used to improve comparability of the treatment and control groups, given known differences in social vulnerability between FQHC communities and the general U.S. population. Moreover, we restricted the analysis to census tracts in counties that contained at least 1 FQHC, further improving comparability by examining census tracts within the same county environment. Each census tract was treated as a unique panel member with time indexed by year; census tracts were then paired with annual statistics for the number of jobs and establishments in each census tract. Census tracts in the sample represented 1,011 distinct counties from all 50 United States, compared to 1,995 counties that did not contain an FQHC and were not included.

### Main measures

Propensity score matching was conducted using the Center for Disease Control and Prevention’s Social Vulnerability Index (SVI) 2010 Estimates. The SVI is a cohesive measure composed of 15 U.S. census variables to help identify communities that may be at risk for hardship during a variety of large-scale disasters, such as heat wave [17], hurricane [18], the September 11 attack on the World Trade Center [19], the opiate epidemic [20], and others. We theorized that the SVI may also be useful in assessing vulnerability to economic recessions, given substantial literature on disparate economic recovery within socially disadvantaged communities [7, 9]. SVI indicators include poverty, unemployment, per capita income, no high school diploma, age 65 years or older, age 17 years or younger, single-parent households, disability, minority racial/ethnic composition, limited English language proficiency, group quarters living arrangements, multi-unit structures, mobile homes, crowding, and vehicle access. Of note, disability was not included in the SVI’s 2010 Estimates, as this variable was added at a later date, and was therefore not included in this analysis.

The primary independent measure of interest was ARRA funding to a U.S. FQHC in 2009. FQHC funding data were provided by the Health Resources & Services Administration and included a list of all FQHCs that received an ARRA capital development grant. Analysis was completed at the site level, with many FQHCs having multiple site addresses. Job change data were obtained from the Longitudinal Employer-Household Dynamics (LEHD) [21] survey and calculated as an annual rate per 1,000 population based on U.S. Bureau of Labor Statistics convention. We used the Residence Area Characteristic (RAC) data files to measure job growth or loss among residents of each FQHC community.

We additionally paired each FQHC’s census tract to the National Neighborhood Data Archive (NaNDA) [22], which provides data for all open and operating establishments at the census tract level. We examined 4 theoretically-relevant establishment types available at the census tract level, which included: healthcare services [23], eating or drinking places [24], retail establishments [25], and grocery stores [26]. Establishment data in the NaNDA are drawn from the National Establishment Time Series (NETS), which provides longitudinal, geocoded data on all business, non-profit and government establishments, updated annually. Establishment change statistics were calculated as an annual rate per 10,000 population based on U.S. Small Business Administration convention [27]. For both job and establishment statistics, we excluded census tracts with a population less than 350 people, which comprised the bottom 1% of census tracts. This eliminated outlier rates that were artificially inflated due to low population counts.

### Statistical analysis

Longitudinal panel analyses were used to implement fixed effects regression at the census tract level. Total jobs were modeled as a function of the interaction between ARRA health center funding status (treatment vs. control) and year, to compare changes in the rate of jobs and establishments recovery between communities that received ARRA health center funding and similar county census tracts. This equation takes on the form: y_it_ = a + x_i_β_a_ + u_it_β_u_ + x_i_u_it_β_xu_ + v_i_ + e_it_, whereby y_it_ is the jobs or establishments rate for census tract i at time t, x_i_ is receipt of ARRA funding for tract i, and u_it_ is the year of observation for census tract i at time t. Receipt of ARRA funding (x_i_) was a time invariant “treatment” and was therefore interacted with a fixed effect for time (u_it_) to estimate the difference in treatment vs. control groups over time.

Propensity score weights were estimated using logistic regression and inverse probability weighting, modeling all 14 variables in the 2010 Social Vulnerability Index (see ‘Main Measures’ above), which did not include disability as previously described. Propensity score weights were then applied to the aforementioned equation. Treatment effects were calculated as the average treatment effect on the treated.

## Results

Of the 50,381 census tracts from 1,011 counties included in the analysis, 2,223 census tracts contained at least one FQHC site that received ARRA health center funding. As expected, FQHC tracts had significantly higher social vulnerability across all SVI measures (Table 1) relative to non-FQHC tracts. Notable among these, a higher proportion of FQHC tracts had an extreme poverty designation (≥ 40% residents living below the federal poverty level; 11.6% vs. 5.4%), > 10% unemployed residents (45.4% vs. 30.3%), > 10% residents with no high school diploma (88.1% vs. 59.0%), > 10% single parent households (70.8% vs. 53.1%), and > 50% minority racial/ethnic composition (48.1% vs. 36.3%).

**Table 1:** Characteristics of census tracts, American community survey 2010 (5-year estimates)

Social Vulnerability Index Measure	Total N = 50,381	Non-FQHC Census Tracts n = 48,158	FQHC Census Tracts n = 2,223	*P*-value
	No. (%)	No. (%)	No. (%)	
Total residents				
< 2000	4,822 (9.6)	4,636 (9.6)	186 (8.4)	
2000–3999	20,216 (40.1)	19,332 (40.1)	884 (39.8)	
4000–5999	17,098 (33.9)	16,335 (33.9)	763 (34.3)	
6000+	8,245 (16.4)	7,855 (16.3)	390 (17.5)	0.13
Total housing units				
< 1000	7,286 (14.5)	7,048 (14.6)	238 (10.7)	
1000–1999	25,900 (51.4)	24,774 (51.4)	1,126 (50.7)	
2000 or more	17,195 (34.1)	16,336 (33.9)	859 (38.6)	< 0.001
Persons living below FPL^a^				
Less than 10%	22,111 (43.9)	21,794 (45.3)	317 (14.3)	
10–19%	14,319 (28.4)	13,565 (28.2)	754 (33.9)	
20–39%	11,072 (22.0)	10, 177 (21.1)	895 (40.3)	
40% or more (extreme poverty)	2,879 (5.7)	2,622 (5.4)	257 (11.6)	< 0.001
Unemployment rate > 10%	15,606 (31.0)	14,597 (30.3)	1,009 (45.4)	< 0.001
Per capita income >$25,000	24,019 (47.8)	23,588 (49.2)	431 (19.4)	< 0.001
No high school diploma > 10%	30,379 (60.3)	28,420 (59.0)	1,959 (88.1)	< 0.001
Persons aged ≥ 65 years				
Less than 10%	17,603 (34.9)	16,890 (35.1)	713 (32.1)	
10–19%	27,162 (53.9)	25,904 (53.8)	1,258 (56.6)	
20% or more	5,616 (11.2)	5,364 (11.1)	252 (11.3)	0.01
Persons aged ≤ 17 years				
20% or more	37,727 (74.9)	36,006 (74.8)	1,721 (77.4)	0.02
Single parent households > 10%	27,138 (53.9)	25,565 (53.1)	1,573 (70.8)	< 0.001
Minority racial/ethnic composition > 50%	18,530 (36.8)	17,460 (36.3)	1,070 (48.1)	< 0.001
Limited English proficiency > 10%	9,389 (18.6)	8,842 (18.4)	547 (24.6)	< 0.001
Persons living in Group Quarters^b^				
Less than 1%	36,038 (71.5)	34,891 (72.5)	1,147 (51.6)	
1–4%	9,426 (18.7)	8,761 (18.2)	665 (29.9)	
5% or more	4,917 (9.8)	4,506 (9.4)	411 (18.5)	< 0.001
Multiunit housing^f^ >10%	19,764 (39.2)	18,963 (39.4)	801 (36.0)	0.002
Crowding^c^ >10%	5,548 (11.0)	5,215 (10.8)	333 (15.0)	< 0.001
Mobile homes > 10%	8,605 (17.1)	7,808 (16.2)	797 (35.9)	< 0.001
No vehicle access				
Less than 10%	33,829 (67.2)	32,734 (68.0)	1,095 (49.3)	
10–19%	8,862 (17.6)	8,310 (17.3)	552 (24.8)	
20% or more	7,690 (15.3)	7,114 (14.8)	576 (25.9)	< 0.001

^a^‘FPL’ indicates federal poverty level. ^b^‘Group quarters’ includes all institutional and non-institutional living facilities not classified as housing units. ^c^’Crowding’ indicates housing units with more than 1 person per room

On average across census tracts, jobs grew at an annual rate of 3.84 jobs per 1,000 population (95% CI: 3.62, 4.06; Table 2) over the study period. In unweighted comparative analyses, job growth in census tracts containing ARRA-funded FQHCs were statistically no different than remaining census tracts in the same county. However, in propensity-score weighted analyses comparing census tracts containing ARRA-funded FQHCs to those with similar SVI indicators, jobs grew at a higher annual rate of 4.34 jobs per 1,000 (95% CI: 2.56, 6.12). Although job growth in weighted analyses was distributed across sectors, the largest gains were in healthcare and social assistance (0.39 jobs per 1,000; 95% CI: 0.11, 0.67) and accommodation and food services (0.41 jobs per 1,000; 95% CI: 0.22, 0.60).

**Table 2:** Annual Change in Number of Jobs 2008–2012 in FQHC Census Tracts Relative to Remaining County Census Tracts

**Jobs per 1,000** *N* = 50,102^b^	Overall Change (95% CI)	Unweighted Model		Propensity Score Weighted Model^a^	
		Relative Change (95% CI)	*P*-value	Relative Change (95% CI)	*P*-value
**Total Jobs**	3.84 (3.62, 4.06)	-0.05 (-1.12, 1.01)	0.92	4.34 (2.56, 6.12)	< 0.001
**Job Type (NAICS Sector)**					
Retail trade (NAICS 44–45)	0.10 (0.07, 0.13)	-0.05 (-0.19, 0.10)	0.53	0.35 (0.13, 0.58)	0.002
Real estate, rental and leasing (NAICS 53)	-0.08 (-0.09, -0.08)	0.02 (-0.01, 0.04)	0.25	0.06 (0.03, 0.10)	0.001
Professional, scientific, and technical (NAICS 54)	0.48 (0.46, 0.50)	-0.21 (-0.31, -0.11)	< 0.001	0.36 (0.22, 0.49)	< 0.001
Healthcare and social assistance (NAICS 62)	2.04 (2.01, 2.08)	-0.12 (-0.28, 0.05)	0.17	0.39 (0.11, 0.67)	0.006
Arts, entertainment, and recreation (NAICS 71)	0.07 (0.06, 0.08)	-0.02 (-0.05, 0.01)	0.24	0.09 (0.03, 0.15)	0.002
Accommodation and food services (NAICS 72)	0.59 (0.57, 0.62)	0.12 (-0.01, 0.24)	0.07	0.41 (0.22, 0.60)	< 0.001

^a^Propensity scores were estimated using logistic regression; models included all 14 variables in the CDC’s 2010 Social Vulnerability Index, including poverty level, unemployment, per capita income, educational attainment, age 65 years or older, age 17 years or younger, single parent households, minority racial/ethnic composition, limited English proficiency, residence in group quarters, multiunit housing, crowding, mobile homes, and vehicle access. ^b^Total number of census tracts reflects inclusion of only census tracts with a total population greater than 350 people

On average across census tracts, establishments grew at an annual rate of 0.23 establishments per 10,000 population (95% CI: 0.19, 0.26; Table 3) over the study period. In unweighted analyses, establishments declined in census tracts containing ARRA-funded FQHCs at an annual rate of -2.42 establishments per 10,000 (95% CI: -2.60, -2.24). In propensity-score weighted analyses, these losses were more limited but remained significant (-1.39 establishments per 10,000; 95% CI: -1.74, -1.04). Losses were primarily in the non-healthcare sector (-1.35 establishments per 10,000; 95% CI: -1.68, -1.02), with retail establishments comprising, on average, 57.7% of the non-healthcare establishments that closed (data not shown). No significant changes were observed for healthcare establishments.

**Table 3:** Annual Change in Number of Establishments 2008–2012 in FQHC Census Tracts Relative to Remaining County Census Tracts

**Establishments per 10,000** *N* = 50,090^b^	Overall Change (95% CI)	Unweighted Model		Propensity Score Weighted Model^a^	
		Relative Change (95% CI)	*P*-value	Relative Change (95% CI)	*P*-value
**Total Establishments**	0.23 (0.19, 0.26)	-2.42 (-2.60, -2.24)	< 0.001	-1.39 (-1.74, -1.04)	< 0.001
Non-Healthcare Establishments^c^	-0.11 (-0.15, -0.08)	-2.24 (-2.41, -2.07)	< 0.001	-1.35 (-1.68, -1.02)	< 0.001
Healthcare Establishments^d^	0.34 (0.33, 0.35)	-0.18 (-0.23, -0.13)	< 0.001	-0.04 (-0.12, 0.04)	0.35

^a^Propensity scores were estimated using logistic regression; models included all 14 variables in the CDC’s 2010 Social Vulnerability Index, including poverty level, unemployment, per capita income, educational attainment, age 65 years or older, age 17 years or younger, single parent households, minority racial/ethnic composition, limited English proficiency, residence in group quarters, multiunit housing, crowding, mobile homes, and vehicle access. ^b^Total number of census tracts reflects inclusion of only census tracts with a total population greater than 350 people. ^c^Non-Healthcare Establishments included eating or drinking places, retail establishments, and grocery stores. ^d^Healthcare Establishments included ambulatory care centers, diagnostic labs, home health services, hospitals, nursing and residential facilities, pharmacies, optical services, and other miscellaneous healthcare establishments

## Discussion

In this longitudinal panel study of FQHCs that received 2009 ARRA funding in response to the Great Recession, we observed mixed findings with respect to local jobs and establishments recovery. Local jobs recovery in FQHC communities was significantly better than in a matched cohort with similar social vulnerability. In these analyses, we observed a 13% higher relative rate of job growth in matched census tracts compared to the overall rate. It is also notable that in unweighted analyses, overall jobs recovery in FQHC communities was no different than the average rate for census tracts in their county. This finding is both unexpected and encouraging, since FQHCs are located in some of the most disadvantaged neighborhoods in the U.S., which generally lag behind in jobs recovery after economic downturns.

By contrast, we did not observe establishment growth in FQHC tracts, but rather, ongoing establishment decline in the 4 years following recession. Establishment losses were predominantly comprised of *non-healthcare* establishments, with the majority (57.7%) being retail establishments. We observed no significant decline in *healthcare* establishments during this time period, consistent with prior research suggesting that ARRA funding likely helped healthcare establishments to maintain operations despite financial challenges [13]. It is probable that some healthcare establishments may have been at risk of closure in the absence of stimulus funding, which would have been detrimental to not only the economic vitality of FQHC communities, but importantly, the many health benefits that FQHCs bring to medically underserved populations [28, 29]. Taken together, our study suggests that direct funding to FQHCs may have been an effective strategy to support healthcare establishments and some local jobs recovery in disadvantaged neighborhoods; but other types of business establishments encountered ongoing hardship and decline.

Although we did not observe extended benefits to neighborhood establishments *beyond healthcare*, as we had hypothesized, the results are informative for guiding future healthcare research and policy. FQHCs are intentionally and strategically located in neighborhoods with the highest levels of social vulnerability and poverty; and keeping stimulus funding within those local communities has been difficult for policy makers. There may be a missed opportunity to leverage health centers more effectively during economic downturns. In an analysis by the Brookings Institute, the number of neighborhoods classified as having extreme poverty (e.g., poverty rates ≥ 40%) increased by 45% in the post-recession era from 2010 to 2014, which resulted in 14 million Americans living in extremely poor conditions [8]. Given strong and enduring associations between neighborhood disadvantage and health [30–32], FQHCs have a vested interest in preventing neighborhood decline and its associated health consequences.

One growing movement aims to equip community health centers with the skills to engage in community development [33], the process of empowering communities to improve their economic prospects through sustainable investment in neighborhood resources [34]. Such movements are based in pragmatic recognition that social determinants are critical levers of population health and often most effectively addressed at the community level. For instance, FQHCs have participated in efforts to improve the community resource infrastructure, such as bringing grocery establishments with fresh produce into food deserts—a resource that is often vulnerable to decline in disadvantaged communities during recession [35]. We theorize that multi-sector use of stimulus funding to integrate healthcare and community development strategies may have a broader impact on declining communities. For example, direct stimulus to FQHCs could support health center efforts to address unmet health-related social needs in housing, education, transportation, infrastructure, social services, and economic development.

Our findings may have implications for targeting public health investments in low-income and disadvantaged communities during economic crises, including in the context of devastating health, social, and economic impacts of the COVID-19 pandemic. On April 1, 2021, the American Rescue Plan appropriated more than $6 billion in direct funding to FQHCs [36], over three times the total funding appropriated by the 2009 stimulus. This new funding was specifically intended to support COVID-19 vaccination, testing, and treatment efforts, sustain preventive and primary health care services, and increase capacity of existing health centers (e.g., physical infrastructure, mobile units) [36]. Similar analyses of 2021 funding efforts should be undertaken in the coming years. However, targeted cross-sector approaches [37, 38], such as integrated community development strategies (e.g., training and employment of local residents) and programs to address the upstream determinants of health (e.g., medical housing programs), may be needed to effectively support local communities at risk for decline.

This study has several limitations. First, the period immediately following the Great Recession was a dynamic time associated with many changes and interventions; there are likely confounders that we were unable to control for. However, we used quasi-experimental methods to compare census tracts (i.e., a relatively small geographical unit) of ARRA-funded FQHCs with a propensity score-matched control group. Moreover, by applying fixed effects to a longitudinal panel, the only relevant confounders would be time-varying confounders; and our review of the literature did not reveal any additional national policies, specifically targeted to a subset of disadvantaged neighborhoods, that may have altered the results.

Second, the study period was limited to 2008–2012, because a second stimulus to FQHCs was appropriated as part of the 2011 Affordable Care Act (ACA) [3]. Although we considered ending the study period in 2011, a large proportion of the ACA funding was withheld by Congress through 2012. In sensitivity analyses using a shorter study period (2008–2011), results remained unchanged: local jobs recovery in FQHC communities was 5.0 jobs per 1,000 population higher (95% CI: 3.2, 6.9) than a matched cohort with comparable social vulnerability.

Third, we were unable to examine spatial spillover due to the implementation of quasi-experimental methods. However, our interest was in the recovery of each FQHC’s local community (i.e., FQHCs tend to serve relatively small geographic areas), rather than economic spillover into higher-income communities. Future work should incorporate spatial lag to examine the spread of economic vitality to surrounding communities. Fourth, we could not compare census tracts containing funded FQHCs to non-funded FQHCs, because the majority of U.S. FQHCs received ARRA funding. However, we used propensity-score matching to compare census tracts containing funded FQHCs to those with similar social vulnerability in the same county, which minimized confounding due to local differences in policy and economic recovery.

Fifth, the SVI is a measure that captures various dimensions of the economic and social environment, but does not fully capture the experiences of persons in communities. Moreover, it was designed to measure a community’s vulnerability to a wide range of external stressors, but was not specifically designed to measure vulnerability to economic stressors such as recession. It is possible that more specific measures pertaining to economic disasters will be available in the future.

Finally, we did not examine the amount of funding awarded to each FQHC or the distribution of funds across multiple sites, which may bias our study toward the null hypothesis. Although HRSA did track the total amount of funding provided to each FQHC, there were limited data on how funds were used. Specific details about the amount of funding used for each project or site would have more accurately reflected the total neighborhood investment and made our study more amenable to a dollar-based analysis. Moreover, variability in the financial health of each FQHC may have impacted the total amount requested by each FQHC, which would not be adequately reflected in a dollar-based analysis.

## Conclusions

We found that the 2009 ARRA health center stimulus was associated with a higher rate of jobs recovery in FQHC tracts relative to a propensity score-matched control group. Even in unweighted models, those that received ARRA funding fared no worse than the remaining tracts in their county—a notable finding, considering the relative disadvantage of FQHC communities. FQHC tracts in our sample had more than twice the number of census tracts qualifying as having extreme poverty relative to non-FQHC tracts. By contrast, we observed no relative improvement in overall establishments recovery, and even observed persistent decline for non-healthcare establishments. Findings suggest that direct funding to health centers may be an effective strategy to support local healthcare establishments and some jobs recovery during economic hardship. However, multidimensional funding strategies, that additionally target upstream determinants of health, may be needed to broaden the scope of future investments and address uneven recovery after recession.

### Electronic supplementary material

Below is the link to the electronic supplementary material.

Supplementary Material 1

## Data Availability

The datasets used and/or analyzed during the current study are not publicly available but can be made available from the corresponding author on reasonable request.
