# Supplementary material for: Impact of 2009 American Recovery and Reinvestment Act (ARRA) health center investments on disadvantaged neighborhoods after recession
Source: Health Econ Rev. 2024 Jan 31;14:9. doi: 10.1186/s13561-024-00482-x (PMC10829270; doi:10.1186/s13561-024-00482-x)
Supplement: Supplementary file 1 — Supplementary Material 1 [file 13561_2024_482_MOESM1_ESM.docx]

**Supplemental Table**. Sources of Data and Main Measures

| **Source of Data** | **Measure**^a^ | **Description** |
| --- | --- | --- |
| Center for Disease Control and Prevention (CDC) | Social Vulnerability Index (SVI), publicly available at: https://www.atsdr.cdc.gov/placeandhealth/svi/index.html | Propensity score weights were based on SVI indicators, including poverty, unemployment, per capita income, no high school diploma, age 65 years or older, age 17 years or younger, single-parent households, minority racial/ethnic composition, limited English language proficiency, group quarters living arrangements, multi-unit structures, mobile homes, crowding, and vehicle access. |
| Health Resources & Services Administration (HRSA) | Receipt of an ARRA Capital Development Grant (binary), provided by HRSA | Primary independent variable of interest (receipt or no receipt of funding) |
| Longitudinal Employer-Household Dynamics (LEHD) | Jobs per 1,000 population, publicly available at: https://lehd.ces.census.gov/data/ | Primary dependent variable of interest:  1) Total jobs  2) Jobs by sector,^b^ including retail trade (NAICS 44-45), real estate, rental and leasing (NAICS 53), professional, scientific and technical (NAICS 54), healthcare and social assistance (NAICS 62), arts, entertainment and recreation (NAICS 71), and accommodation and food services (NAICS 72). |
| National Neighborhood Data Archive (NaNDA) | Establishments per 10,000 population, publicly available at: https://nanda.isr.umich.edu | Secondary dependent variable of interest:  1) Total establishments  2) Establishments by sector, including (a) healthcare (ambulatory care centers, diagnostic labs, home health services, hospitals, nursing and residential facilities, pharmacies, optical services, and other miscellaneous healthcare establishments) and (b) non-healthcare (eating or drinking places, retail establishments, and grocery stores). |

^a^All measures were analyzed at the census tract level.

^b^NAICS denotes North American Industry Classification System, the standard used by Federal statistical agencies in classifying business establishments for the purpose of collecting, analyzing, and publishing statistical data related to the U.S. business economy.
